# C-752-08. Skin Graft Loss Risk Estimator: An Interpretable Machine Learning Tool for Burn Patient Assessment

**DOI:** 10.1093/jbcr/irag033.021

**Published:** 2026-04-06

**Authors:** Francesco M Egro, Abbas M Hassan, Hilary Liu, Leigh J Spera

**Affiliations:** University of Pittsburgh Medical Center, Pittsburgh, PA; Indiana University, Indianapolis, IN; University of Pittsburgh Medical Center, Pittsburgh, PA; Indiana University/Eskenazi Health, Indianapolis, IN

## Abstract

**Introduction:**

Skin graft failure is a significant complication in burn care that prolongs recovery, compromises surgical outcomes, and necessitates repeat operations. Accurate risk assessment is challenging due to a complex interplay of patient and injury factors, underscoring the need for objective, data-driven predictive tools. The objective of this study is to develop and validate an interpretable machine learning (ML) tool to provide individualized, data-driven risk assessment for skin graft loss in burn patients.

**Methods:**

A cohort study of patients who had autologous skin grafting from the American Burn Association Burn Care Quality Platform was conducted from 2013-2022. A Random Forest ML model was developed and validated on independent training (70%), validation (15%), and testing (15%) sets. Model performance was assessed using the area under the receiver operating characteristic curve (AUC-ROC).

**Results:**

Of 286 479 patients during the study period, 24 046 underwent autologous skin grafting with skin graft loss incidence of 2.0%. ML demonstrated good discrimination with AUC-ROC of 0.794 (Fig. 1). Burns ≥40% total body burn surface area (OR 8.16, *p*<.001), history of alcohol abuse (OR 1.86, *p*<.001), active tobacco use (OR 1.45, *p*=.004), age > 60 years (OR 1.70, *p*=.013), hypertension (OR 1.52, *p*=.003), diabetes mellitus (OR 1.45, *p*=.030) and contact burns (OR 1.70, *p*=.006) emerged as independent predictors of graft loss. The model was translated into an interactive, interpretable web-based clinical tool: https://rb.gy/2n66sp (Fig. 2).

**Conclusions:**

The Skin Graft Loss Risk Estimator provides a transparent, interpretable and personalized risk assessment of skin graft loss to enhance clinical judgment, facilitate effective patient counseling and surgical planning in burn care.

**Applicability of Research to Practice:**

The Skin Graft Loss Risk Estimator advances research practice by standardizing risk quantification, enabling stratification of high-risk patients in trials, and clarifying the impact of comorbidities and burn characteristics on graft outcomes. Its interpretable design allows investigators to test targeted interventions and validate findings across multicenter studies.

**Funding for the Study:**

N/A.

